# Benefit Design and Potential Trade-offs of Medicare Advantage Affinity Plans for Asian Beneficiaries

**DOI:** 10.1001/jamanetworkopen.2025.48028

**Published:** 2025-12-16

**Authors:** Yanlei Ma, Pasha Hamed, Rebekah I. Stein, Nishmi Abeyweera, E. John Orav, Thomas C. Tsai, Jose F. Figueroa

**Affiliations:** 1 Department of Health Policy and Management, Harvard T.H. Chan School of Public Health, Boston, Massachusetts; 2 Harvard Medical School, Boston, Massachusetts; 3 Department of Medicine, Brigham and Women’s Hospital, Boston, Massachusetts; 4 Department of Surgery, Brigham and Women’s Hospital, Boston, Massachusetts

## Abstract

**Importance:**

As Medicare Advantage (MA) continues to expand, an increasing number of MA plans are marketed to specific affinity groups, including Asian Medicare beneficiaries in the US. Little is known about the potential trade-offs of these emerging Asian-oriented affinity plans.

**Objectives:**

To evaluate the prevalence of MA Asian-oriented affinity plans and the characteristics of their Asian beneficiaries, to understand the differences in plan-benefit design between these affinity plans and other MA plans, and to compare the breadth of MA physician networks of Asian-oriented affinity plans vs other MA plans.

**Design, Setting, and Participants:**

This cross-sectional study used 2023 national Medicare data to identify Asian-oriented affinity plans. MA plans were identified as Asian-oriented affinity plans if the difference in Asian enrollment between the plan and its service area exceeded the 99th percentile of the Box-Cox–transformed normal distribution. When comparing Asian-oriented affinity plans with other MA plans, the sample was restricted to states where Asian-oriented affinity plans were offered. Data analyses were performed from June 2024 to June 2025.

**Exposure:**

Asian-oriented affinity plan classification.

**Main Outcomes and Measures:**

Cost-sharing, supplemental benefits, Medicare Star Ratings, and network breadth. Enrollee-weighted, adjusted regression models were used to assess differences in plan-benefit design, Medicare Star Ratings, and network breadth between Asian-oriented affinity plans and other MA plans.

**Results:**

The sample included 4224 MA plans in 2023, of which 27 were identified as Asian-oriented affinity plans. These 27 plans were offered in California, New York, Texas, and Massachusetts and enrolled 16.1% (109 906 of 684 764) of Asian beneficiaries in these states. Asian enrollees in these plans (mean [SD] age, 73.0 [7.19] years; 57 729 females [52.5%]) were more likely to be older, male, without disability, and dually eligible for Medicaid-Medicare benefits. Compared with other MA plans, Asian-oriented affinity plans had a higher likelihood of $0 Part C premiums (adjusted difference, 10.8 [95% CI, 10.8-10.9] percentage points), Part B premium reductions (adjusted difference, 6.7 [95% CI, 6.5-6.9] percentage points), and lower monthly Part D premiums (adjusted difference, −$7.18 [95% CI, −$14.24 to −$0.12]). Asian-oriented affinity plans were more likely than other MA plans to provide culturally relevant benefits, including acupuncture (adjusted difference, 23.2 [95% CI, 23.0-23.4] percentage points) and alternative therapies (adjusted difference, 4.8 [95% CI, 4.7-5.0] percentage points). However, Asian-oriented affinity plans were less likely to cover annual physical examinations (adjusted difference, −41.7 [95% CI, −41.9 to −41.5] percentage points), had lower Medicare Star Ratings, and had narrower physician networks compared with other MA plans.

**Conclusions and Relevance:**

In this cross-sectional study, while MA Asian-oriented affinity plans offered more culturally relevant benefits and favorable premiums, they came with important trade-offs, including narrower physician networks, lower Medicare Star Ratings, and reduced coverage of certain traditional benefits. Enrollment growth and performance of Asian-oriented affinity plans should be closely monitored to ensure that they address the health care needs of Asian beneficiaries.

## Key Points

**Question:**

What are the characteristics and potential trade-offs—related to cultural relevance, benefit coverage, and network restrictions—of Medicare Advantage (MA) affinity plans that disproportionately enroll the Asian population in the US?

**Findings:**

In this cross-sectional study of 4224 MA plans in 2023, 27 were identified as Asian-oriented affinity plans offered in California, New York, Texas, and Massachusetts, collectively enrolling 16.1% of Asian MA beneficiaries in these states. These affinity plans, compared with other MA plans, offered lower premiums and culturally relevant benefits but were less likely to cover traditional benefits, achieved lower Medicare Star Ratings, and had narrower physician networks.

**Meaning:**

The enrollment growth and performance of Asian-oriented affinity plans should be closely monitored to ensure they are meeting Asian beneficiaries’ health care needs.

## Teaser

This cross-sectional study characterizes the enrollees, coverage, networks, and performance of Medicare Advantage affinity plans tailored for Asian Medicare beneficiaries in the US.

## Introduction

Medicare beneficiaries are increasingly enrolling in Medicare Advantage (MA) plans. In 2024, 54% of the Medicare population enrolled in MA over traditional Medicare.^1^ As MA continues to expand, an increasing number of MA plans are being marketed and tailored to specific populations based on their culture, gender, veteran status, and race and ethnicity.^2,3,4^ Recent evidence shows that enrollment in these specialized MA affinity plans has been steadily growing, including among plans designed for Asian beneficiaries in the US.^5^

MA affinity plans have the potential to address the unique health care needs of specific populations by offering customized benefits design, culturally competent care, and improved language accessibility.^3,4,6^ However, to date, there is limited evidence documenting the extent to which affinity plans achieve these goals or whether enrolling in these plans comes with important trade-offs. There is growing concern that affinity plans may primarily serve as a marketing strategy to increase enrollment and maximize profitability without delivering the purported benefits or improving quality of care.^2,3,7^ This phenomenon is particularly concerning for the Asian population, as studies have shown that Asian beneficiaries tend to underutilize Medicare services and incur lower health care expenditures, on average,^8,9,10^ making them potentially attractive to MA affinity plans seeking to maximize profits. Low utilization among Asian beneficiaries 65 years or older might be partly explained by patterns of prolonged stays overseas, particularly in their countries of origin where living costs are lower and familial support is stronger, resulting in extended periods without incurring any medical services paid by MA plans.^11,12,13^

Despite the increasing prevalence of MA Asian-oriented affinity plans, to our knowledge, little is known about the characteristics these plans use to attract Asian enrollees—specifically, plan-benefit design and physician networks—or whether enrollment comes with trade-offs for beneficiaries. Therefore, using national Medicare data, we conducted this study with the following key objectives. First, we sought to evaluate the prevalence of MA Asian-oriented affinity plans and the characteristics of their Asian beneficiaries. Second, we aimed to understand the differences in plan-benefit design, including premiums, deductibles and supplemental benefits, and Medicare Star Ratings, between Asian-oriented affinity plans and other MA plans. Third, we aimed to compare the breadth of MA physician networks of Asian-oriented affinity plans vs other MA plans.

## Methods

The Harvard T.H. Chan School of Public Health Institutional Review Board approved this cross-sectional study and waived the informed consent requirement because the data used were deidentified. We followed the Strengthening the Reporting of Observational Studies in Epidemiology (STROBE) reporting guideline.

### Data Sources

We used the 2023 Medicare Master Beneficiary Summary Files to obtain MA plan enrollment and beneficiary demographic information^14^ as well as the Plan Characteristics Files and MA Plan Directory to capture plan type, service area, and tax status.^15,16^ To compare plan cost-sharing structure and supplemental benefits between Asian-oriented affinity plans and other MA plans, we used the Centers for Medicare and Medicaid Services (CMS) Landscape Files^17^ and Plan Benefit Package files.^18^ To compare plan performance, we used Medicare Star Ratings data.^19^ To compare enrollee health risk, we used the plan-level risk score from CMS Plan Payment Data.^20^ Additionally, to identify physicians participating in each network and to compare physician networks between Asian-oriented affinity plans and other MA plans, we used the 2023 Ideon provider network data, which include a list of clinicians and facilities participating in each MA network based on scraping online directories.^21^ We treated the analysis with Ideon data as exploratory given the potential inaccuracies of the directories.^22,23^

### Identifying Asian-Oriented Affinity Plans

In the primary analysis, Asian beneficiaries were identified using the Research Triangle Institute (RTI International) race and ethnicity code for Asian American and Pacific Islander individuals, which uses a modified algorithm on self-reported data.^14^ Prior research has shown that such race and ethnicity code has high specificity but somewhat lower sensitivity when compared with self-reported race and ethnicity survey data.^24^

To identify Asian-oriented affinity plans, we first calculated for each MA plan (1) the percentage of Asian enrollees within the plan and (2) the percentage of Asian MA enrollees within the plan’s service area. We then transformed the difference between these 2 percentages to a normal distribution using the Box-Cox transformation. We chose this approach as our primary method because the Box-Cox transformation reduces skewness and does not assume the underlying differences are normally distributed. An MA plan was classified as an Asian-oriented affinity plan if the transformed difference between the plan’s Asian enrollment share and the Asian MA enrollment share in its service area exceeded the 99th percentile.^25^

To assess the robustness of our approach, we explored 2 alternative methods of identifying Asian-oriented affinity plans. First, we classified an MA plan as an Asian-oriented affinity plan if the untransformed difference between its Asian enrollment and the Asian MA enrollment in its service area exceeded 3 SDs, which approximates the 99th percentile of the deviation from the Asian MA enrollment in the plan’s service area. Second, we used the Medicare Bayesian Improved Surname Geocoding algorithm^26^ instead of the RTI International race and ethnicity code to identify Asian beneficiaries and applied the same methods as the primary approach to classify Asian-oriented affinity plans. Both alternative methods identified a similar set of Asian-oriented affinity plans as did our primary approach (eTable 1 in Supplement 1).

### Sample

The study sample included MA plans with both Medicare Part C and Part D coverage and with nonzero Medicare beneficiary enrollment in 2023. Regional preferred provider organizations, Program of All-Inclusive Care for the Elderly plans, employer plans, cost plans, Medicare Medical Savings Account plans, stand-alone drug plans, and fall-back contracts were excluded from the analyses. MA plans with fewer than 100 enrollees were also excluded from the analyses to avoid spurious classifications of Asian enrollment due to small sample sizes. When comparing between Asian-oriented affinity plans and other MA plans, we further limited the sample to MA plans offered in states where Asian-oriented affinity plans were available and to beneficiaries residing in those states.

### Outcomes

The study focused on 4 outcomes. First, we examined cost-sharing measures, including the Part C premium amount, whether a plan has a $0 Part C premium, Part D premium amount, Part D deductible amount, whether a plan offers a Part B premium reduction, and the Part B premium reduction amount. Second, we examined the offering of supplemental benefits as binary measures, including whether a plan offers preventive and comprehensive dental services, vision services and eyewear, hearing aids and examinations, transportation services, over-the-counter coverage, acupuncture, chiropractic services, podiatry services, annual physical examination, alternative therapies, remote-access technologies, in-home support services, and worldwide emergency or urgent care. Third, we examined plan Medicare contract Star Rating performance, including Part C, Part D, and overall Medicare Star Ratings. Fourth, as an exploratory analysis, we measured network breadth as the enrollment-weighted proportion of physicians within a plan’s service area that participated in the plan’s network.^27^ Specifically, we first calculated the proportion of in-network physicians at the plan county level. We then aggregated these values across all counties within the plan’s service area weighted by county-level enrollment. Similar to prior work, we categorized network breadth into 5 groups: extra small (<10%), small (10% to <25%), medium (25% to <40%), large (40% to <60%), and extra large (≥60%).^28^

### Statistical Analysis

We conducted 4 sets of analyses. First, we identified Asian-oriented affinity plans in 2023 by comparing each MA plan’s Asian enrollment percentage with the prevalence of Asian MA enrollees in that plan’s service area. For each Asian-oriented affinity plan, we also reviewed the plan website and confirmed the presence of any Asian-focused marketing content (eg, coverage of acupuncture, herbal medicine, and Tai Chi classes).

Second, to assess the characteristics of Asian beneficiaries associated with enrollment in Asian-oriented affinity plans, we first reported the unadjusted proportions across beneficiary characteristics between Asian-oriented affinity plans and other MA plans. We then estimated the likelihood of enrollment in Asian-oriented affinity plans using a logistic regression model wherein the dependent variable was whether an Asian beneficiary was enrolled in an Asian-oriented affinity plan and the explanatory variables included beneficiaries’ nominal age categories (<55, 55-64, 65-79, and ≥80 years), sex, original reason for Medicare entitlement, Medicare-Medicaid dual eligibility, and state of residence. We reported adjusted odds ratios along with 95% CIs.

Third, we compared cost-sharing, supplemental benefits, Medicare contract Star Rating performance, and enrollee health risk score between Asian-oriented affinity plans and other MA plans. For unadjusted comparisons, we reported the mean of each outcome measure separately for Asian-oriented affinity plans and other MA plans. For adjusted comparisons, we estimated separate enrollee-weighted plan-level regression for each outcome measure, where the dependent variable was the outcome measure of interest and the key explanatory variable was whether a plan was an Asian-oriented affinity plan, controlling for plan type, special needs plan status, and state fixed effects. We reported adjusted differences along with 95% CIs.

Fourth, as an exploratory analysis, we compared physician network size between Asian-oriented affinity plans and other MA plans using Ideon provider networks data. For unadjusted comparisons, we reported the proportion of Asian-oriented affinity plans and proportion of other MA plans across the 5 network breadth categories. For adjusted comparisons, we estimated plan-level linear regressions, with network breadth as the dependent variable and an indicator for Asian-oriented affinity plans as the key explanatory variable, controlling for plan type, special needs plan status, and state fixed effects. These comparisons were conducted separately for all physicians, primary care physicians (PCPs), and specialists. Given the high number of Asian-oriented affinity plans offered in California compared with other states, we also conducted stratified analyses for plans offered in California vs plans offered elsewhere. As a sensitivity check, we excluded plans with network breadth in the lowest fifth percentile, as extremely narrow networks may reflect incomplete or inaccurately captured data in the Ideon dataset.

Analyses were performed from June 2024 to June 2025 using SAS, version 9.4 (SAS Institute Inc). The threshold for statistical significance was set at *P* < .05. However, given that multiple outcomes were compared, 95% CIs, rather than *P* values, are reported.

## Results

### Plan and Beneficiary Characteristics

**Figure 1.**
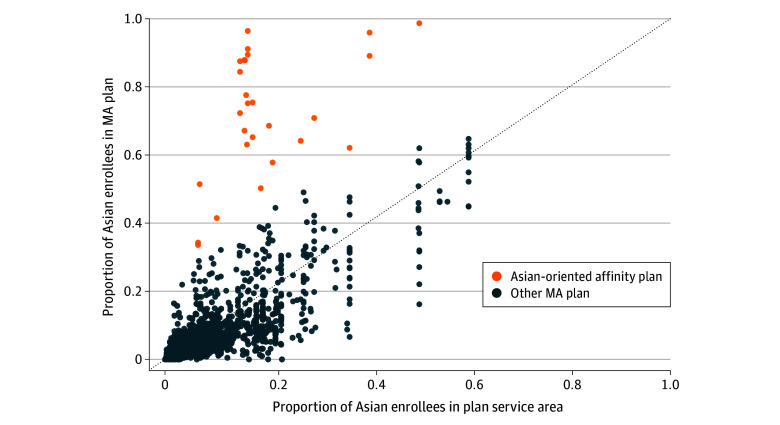
Proportion of Asian Enrollees in Medicare Advantage (MA) Plans Against Asian Enrollees in Plan Service Areas in 2023 The dashed line represents the scenario when the proportion of Asian enrollees in an MA plan equals the proportion of Asian MA enrollees in the plan's service area. An MA plan was classified as an Asian-oriented affinity plan if the difference between the plan’s Asian enrollment and the Asian MA enrollment in its service area exceeded the 99th percentile of the Box-Cox–transformed normal distribution.

The study sample included 4224 MA plans in 2023. Among these MA plans, 27 were identified as Asian-oriented affinity plans, with Asian enrollment ranging between 33.6% and 98.7% of the plan's total enrollment (Figure 1). When applying alternative methods to identify Asian-oriented affinity plans, we found substantial similarities with Asian-oriented affinity plans identified using our primary approach (eTable 1 in Supplement 1).

The 27 Asian-oriented affinity plans were offered in California, New York, Texas, and Massachusetts. While these plans accounted for 2.8% (169 802) of the total MA enrollment in these states (6 256 661), they enrolled 16.1% (109 906) of the Asian MA population (684 764) (Table 1). Compared with Asian enrollees in other MA plans within these states, those enrolled in Asian-oriented affinity plans were more likely to be older, male, without disability, and dually eligible for Medicaid-Medicare benefits (Table 1). These beneficiaries had a mean (SD) age of 73.0 (7.19) years and comprised 57 729 females (52.5%) and 52 177 males (47.5%).

**Table 1.** Characteristics of Asian Beneficiaries Enrolling in Asian-Oriented Affinity Plans vs Other Medicare Advantage Plans in 2023^a^

Characteristic	Asian beneficiaries, No. (%)		AOR for Asian-oriented affinity plan enrollment (95% CI)^b^
	Other MA plans	Asian-oriented affinity plans	
No. of plans	928	27	NA
Total No. of enrollment	6 086 859	169 802	NA
No. of Asian beneficiaries	574 858	109 906	NA
% Asian MA enrollees	9.4	64.7	NA
Age, y			
<55	9542 (1.7)	513 (0.5)	1 [Reference]
55-64	14 301 (2.5)	1566 (1.4)	1.95 (1.76-2.17)
65-79	434 680 (75.6)	88 843 (80.8)	2.30 (2.09-2.53)
≥80	116 335 (20.2)	18 984 (17.3)	1.74 (1.58-1.92)
Sex			
Male	256 930 (44.7)	52 177 (47.5)	1 [Reference]
Female	317 928 (55.3)	57 729 (52.5)	0.88 (0.87-0.89)
Original reason for Medicare entitlement			
OASI	524 498 (91.2)	104 657 (95.2)	1 [Reference]
Disability	48 294 (8.4)	5146 (4.7)	0.58 (0.56-0.60)
ESKD	1566 (0.3)	82 (0.1)	0.33 (0.26-0.41)
Disability and ESKD	500 (0.1)	21 (0.0)	0.24 (0.15-0.37)
Medicare-Medicaid dual eligibility			
Not dually eligible	312 986 (54.4)	50 097 (45.6)	1 [Reference]
Full Medicaid benefits	241 988 (42.1)	55 755 (50.7)	1.25 (1.24-1.27)
Partial Medicaid benefits	19 884 (3.5)	4054 (3.7)	1.33 (1.28-1.38)
Enrollment by geography			
California	344 803 (60.0)	60 608 (55.1)	1 [Reference]
Massachusetts	18 975 (3.3)	6183 (5.6)	1.75 (1.69-1.80)
New York	132 924 (23.1)	36 876 (33.6)	1.42 (1.40-1.44)
Texas	78 156 (13.6)	6239 (5.7)	0.45 (0.44-0.46)

Abbreviations: AOR, adjusted odds ratio; ESKD, end-stage kidney disease; MA, Medicare Advantage; NA, not applicable; OASI, Old-Age and Survivors Insurance.

^a^ Analysis was limited to MA plans offered in California, New York, Texas, and Massachusetts and beneficiaries residing in these states.

^b^ Adjusted ORs were derived from a logistic regression model wherein the dependent variable was whether an Asian beneficiary was enrolled in an Asian-oriented affinity plan and the explanatory variables were beneficiaries’ age, sex, original reason for Medicare entitlement, Medicare-Medicaid dual eligibility, and state of residence.

We confirmed that all Asian-oriented affinity plans identified featured some form of Asian-focused marketing content on their website, highlighting services such as acupuncture, herbal medicine, and Tai Chi classes (eFigure in Supplement 1). Compared with other MA plans in these 4 states, Asian-oriented affinity plans were more likely to be Special Needs Plans (25.4% vs 46.0%) and sponsored by for-profit organizations (72.4% vs 92.6%) (eTable 2 in Supplement 1). In addition, Asian-oriented affinity plans had lower mean enrollee health risk scores than other MA plans (1.09 vs 1.16; adjusted difference, −0.12; 95% CI, −0.20 to −0.04) after accounting for plan type and geography (Table 2).

**Table 2.** Cost-Sharing, Medicare Star Rating Performance, and Enrollee Health Risk for Asian-Oriented Affinity Plans vs Other Medicare Advantage Plans in 2023^a^

	MA plans^b^^,^^c^		Difference	
	Asian-oriented affinity	Other	Unadjusted^b^^,^^c^	Adjusted (95% CI)^c^^,^^d^
Cost-sharing				
Mean Part C premium, $	0.00	3.99	−3.99	−4.10 (−10.71 to 2.52)
Proportion offering $0 Part C premium, percentage point	100.0	89.1	10.9	10.8 (10.8 to 10.9)
Mean Part B premium reduction amount, $	10.26	4.84	5.42	7.33 (−1.35 to 16.01)
Proportion offering Part B premium reduction, percentage point	9.7	5.6	4.1	6.7 (6.5 to 6.9)
Mean Part D premium, $	12.59	13.81	−1.22	−7.18 (−14.24 to −0.12)
Mean Part D deductible amount, $	248.99	168.66	80.33	−8.89 (−55.61 to 37.82)
Medicare contract Star Rating				
Part C	3.19	4.03	−0.84	−0.69 (−0.97 to −0.42)
Part D	3.39	4.05	−0.66	−0.64 (−0.94 to −0.34)
Overall	3.27	4.14	−0.87	−0.73 (−1.04 to −0.42)
Enrollee health risk				
Mean HCC risk score	1.09	1.16	−0.07	−0.12 (−0.20 to −0.04)

Abbreviations: HCC, Hierarchical Condition Category; MA, Medicare Advantage.

^a^ Analysis was limited to MA plans offered in California, New York, Texas, and Massachusetts.

^b^ Values represent unadjusted means.

^c^ Both unadjusted and adjusted analyses are weighted by number of beneficiaries.

^d^ Adjusted differences between Asian-oriented affinity plans and other MA plans were estimated using enrollee-weighted linear regression models. The dependent variable was the outcome measure of interest, and explanatory variables included an indicator for Asian-oriented affinity plans, plan type, special needs plan status, and state fixed effects.

### Cost-Sharing

Asian-oriented affinity plans exhibited different cost-sharing structures compared with other MA plans (Table 2). After accounting for differences in plan type and geography, Asian-oriented affinity plans were more likely to offer $0 Part C premium (adjusted difference, 10.8 [95% CI, 10.8-10.9] percentage points) and Part B premium reductions (adjusted difference, 6.7 [95% CI, 6.5-6.9] percentage points). Additionally, Asian-oriented affinity plans had a significantly lower Part D premiums compared with other MA plans (adjusted difference, −$7.18; 95% CI, −$14.24 to −$0.12) (Table 2).

### Supplemental Benefits

**Figure 2.**
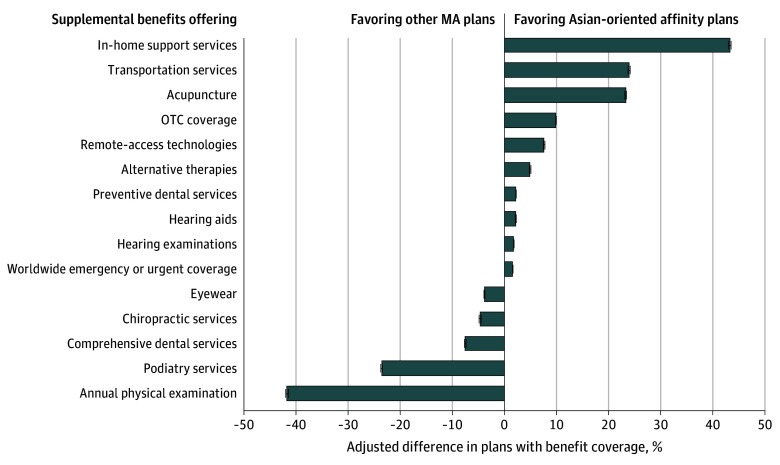
Adjusted Differences in Supplemental Benefit Offerings Between Asian-Oriented Affinity Plans and Other Medicare Advantage (MA) Plans in 2023 Adjusted differences in supplemental benefit coverage were estimated using enrollee-weighted logistic regression models. Analysis was limited to MA plans offered in California, New York, Texas, and Massachusetts. Error bars represent 95% CIs. OTC indicates over the counter.

Asian-oriented affinity plans differed from other MA plans in their supplemental benefits offerings (Figure 2). After adjusting for plan type and geography, Asian-oriented affinity plans were significantly more likely to cover acupuncture (adjusted difference, 23.2 [95% CI, 23.0-23.4] percentage points) and alternative therapies (adjusted difference, 4.8 [95% CI, 4.7-5.0] percentage points) (eTable 3 in Supplement 1). Asian-oriented affinity plans were also more likely than other MA plans to provide in-home support services, transportation services, over-the-counter drug coverage, remote-access technologies, and worldwide emergency or urgent care coverage (eTable 3 in Supplement 1). However, Asian-oriented affinity plans were significantly less likely than other MA plans to offer certain traditional benefits such as annual physical examination (adjusted difference, −41.7 [95% CI, −41.9 to −41.5] percentage points), podiatry services (adjusted difference, −23.6 [95% CI, −23.8 to −23.4] percentage points), and comprehensive dental services (adjusted difference, −7.6 [95% CI, −7.7 to −7.4] percentage points) (eTable 3 in Supplement 1).

### Medicare Star Rating Performance

Asian-oriented affinity plans exhibited lower Medicare contract Star Ratings compared with other MA plans. After adjusting for plan type and geography, Asian-oriented affinity plans were associated with lower Part C (adjusted difference, −0.69; 95% CI, −0.97 to −0.42), Part D (adjusted difference, −0.64; 95% CI, −0.94 to −0.34), and overall (adjusted difference, −0.73; 95% CI, −1.04 to −0.42) Medicare Star Ratings (Table 2). These patterns were consistent across all individual rating domains, with affinity plans scoring lower on preventive care, chronic condition management, member experience, and drug safety measures (eTable 4 in Supplement 1).

### Physician Network Size

**Figure 3.**
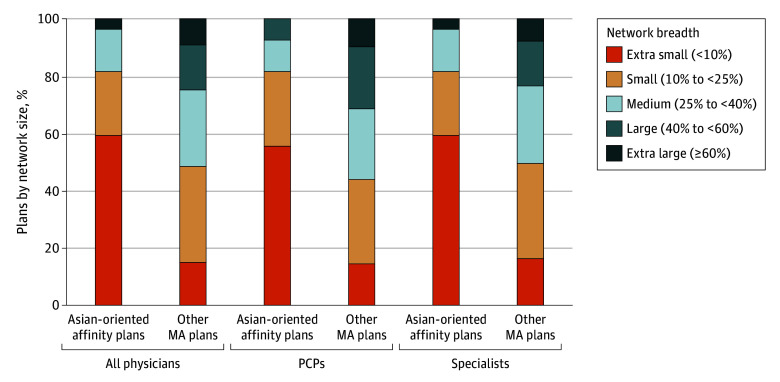
Physician Network Breadth for Asian-Oriented Affinity Plans vs Other Medicare Advantage (MA) Plans in 2023 Network size categories were constructed based on network breadth, which was first calculated at the plan-county level as the proportion of in-network physicians in a county out of the total number of physicians in network for at least 1 plan of analysis in that county. Plan-level network breadth was then calculated as a beneficiary-weighted average of plan-county breadths. PCP indicates primary care physician.

Asian-oriented affinity plans had narrower physician networks than other MA plans. In unadjusted comparisons, more than 80% of Asian-oriented affinity plans (81.5% for both PCPs and specialists) had small or extra small physician networks compared with less than 50% of other MA plans (43.9% for PCPs and 49.5% for specialists) (Figure 3). After adjusting for plan type and geography, there was no significant overall difference in network breadth between Asian-oriented affinity plans and other MA plans. However, when stratified by state, Asian-oriented affinity plans offered in California had significantly narrower networks than other MA plans in the state. Specifically, PCP networks of Asian-oriented affinity plans were 16.0 (95% CI, 7.3 to 24.6) percentage points narrower and specialist networks were 11.6 (95% CI, 4.0 to 19.1) percentage points narrower compared with networks in other MA plans (eTable 5 in Supplement 1). In contrast, we did not observe any differences in network breadth in other states. These results remain qualitatively unchanged when we excluded plans with network breadth in the lowest fifth percentile (eTable 6 in Supplement 1).

## Discussion

In this national study of Asian-focused MA affinity plans, we found that a modest number of plans enrolled 16.1% of Asian MA beneficiaries in California, New York, Texas, and Massachusetts in 2023. Compared with Asian beneficiaries enrolled in other MA plans, those in Asian-oriented affinity plans were more likely to be older, male, without disability, and dually eligible for Medicaid-Medicare benefits. Asian-oriented affinity plans tended to offer more favorable premiums and provide culturally relevant supplemental benefits, such as acupuncture and alternative therapies. However, these plans were less likely to cover certain traditional benefits such as annual physical examinations and comprehensive dental care, achieved lower Medicare Star Ratings, and had narrower physician networks than other MA plans in California, where most Asian-oriented affinity plans operate.

Our findings suggest that Asian-oriented affinity plans are using targeted benefit designs to attract Asian beneficiaries. First, these plans leverage Asian-focused supplemental benefits as a key strategy to appeal to the Asian population. Consistent with their Asian-focused marketing content, these affinity plans were significantly more likely to cover acupuncture and alternative therapies—services that are used at higher rates among the Asian population, particularly individuals with limited English proficiency.^29,30,31^ Second, similar to MA affinity plans for other groups (eg, veterans), Asian-oriented affinity plans offered favorable premiums, including a higher likelihood of $0 Part C premium and Part B premium reductions. Such financial incentives may be particularly appealing to Asian beneficiaries with lower incomes, who may be cost-sensitive and prioritize affordability when selecting an MA plan. Given that some Asian retirees might spend extended periods overseas and are less likely to use Medicare services while outside the US, insurers may expect lower utilization and thus be more willing to offer reduced cost-sharing in these Asian-oriented affinity plans. Additionally, the results suggest that these affinity plans may be selectively targeting lower-risk Asian beneficiaries to enhance profitability.

While Asian-oriented affinity plans provide attractive premiums and benefits, they also come with important trade-offs. A key concern is that many Asian-oriented affinity plans have narrow physician networks, particularly in California, which may limit beneficiaries’ physician choices and increase their financial burden if they need out-of-network care. Given that Asian people historically tend to underuse health care services,^8,9,10^ narrower networks may further exacerbate their barriers to care. Additionally, the majority of Asian-oriented affinity plans do not offer comprehensive annual physical examinations, which could hinder early detection and management of health conditions. Finally, lower Medicare Star Rating performance—although imperfect—serves as a potential proxy measure for clinical processes of care and patient experience, raising concerns that Asian enrollees in these affinity plans may be receiving lower-quality care services than in other MA plans. However, further research is needed to evaluate the performance of these plans on key clinical measures and health outcomes.

While Asian-oriented affinity plans purport to offer certain benefits, it remains unclear to what extent these benefits are actually used. For example, although Asian-oriented affinity plans are substantially more likely to cover in-home support services, studies show that Asian beneficiaries often underuse formal home health services, instead relying on informal family-based care.^32^ Moreover, dual-eligible beneficiaries may be less likely to take up in-home support services since Medicaid already covers home- and community-based services and long-term care support. Therefore, it is possible that supplemental benefits are underused, allowing MA plans to enhance their appeal without incurring actual costs.^33^

Asian-oriented affinity plans are currently concentrated in 4 states with large Asian populations, but their disproportionate enrollment of Asian beneficiaries raises concerns. Many Asian beneficiaries, particularly those with limited health and English literacy, may be selecting these plans based primarily on cultural relevance and lower premiums, without fully understanding the trade-offs in clinician access and benefit coverage. Similarly, dual-eligible Asian beneficiaries may be drawn to these plans for their cultural tailoring and pay little attention to limitations in plan benefits given that Medicaid provides additional coverage. Given the steady enrollment growth of Asian-oriented affinity plans in recent years,^5^ policymakers may want to consider implementing stronger consumer education initiatives to help enrollees make informed choices. Additionally, policymakers may want to increase regulatory oversight to ensure that these plans deliver real value to Asian beneficiaries and are not simply using marketing tactics to increase their profitability. For instance, efforts could focus on monitoring disparities in health care utilization and health outcomes between enrollees of Asian-oriented affinity plan and those in other MA plans to help identify potential gaps in care quality and access.

This study contributes in several ways to the growing body of research on MA affinity plans. To our knowledge, this study was the first to examine affinity plans tailored to Asian beneficiaries, a group experiencing the highest enrollment increase among all affinity plan categories.^5^ Additionally, this study analyzed the supplemental benefits and physician networks of Asian-oriented affinity plans, providing insight into the important trade-offs faced by enrollees in these affinity plans. Finally, our findings align with those of Meiselbach et al,^5^ who reported that, similar to broader category of other affinity plans (including those targeting women, other racial and ethnic minority groups, and the LGBTQAI [lesbian, gay, bisexual, transgender, queer or questioning, asexual or allied, intersex] population), Asian-oriented affinity plans tend to have lower premiums and lower Medicare Star Ratings.

### Limitations

This study has several limitations. First, our classification of Asian-oriented affinity plans was based on an Asian beneficiary enrollment threshold, which may misclassify certain MA plans as Asian-oriented affinity plans even if they were not explicitly marketed toward Asian beneficiaries. It is also possible that some plans with explicit Asian-focused marketing were not identified as Asian-oriented affinity plans because their Asian enrollment did not exceed the threshold, especially those offered in areas with a high proportion of Asian residents. Nevertheless, we confirmed that all Asian-oriented affinity plans had some form of Asian-focused marketing content on their website. Additionally, our sensitivity analyses showed that the findings remained largely unchanged as we varied the threshold or used an alternative race and ethnicity variable. Second, the RTI International race variable, despite including self-reported data for Asian people, has well known limitations. While prior research has shown this variable to have high specificity for Asian people (99.8%) when compared with national surveys that included self-reported data, the variable has lower sensitivity and does not allow the disaggregation of the diverse Asian population into subgroups (eg, Chinese, Japanese, or Korean).^24^ Third, we limited the comparison to plan-benefit design and physician networks, and we did not examine Asian beneficiaries’ use of benefits or access to clinicians due to the lack of comprehensive benefit utilization data available to researchers. Future research could explore whether Asian beneficiaries are actively using Asian-focused benefits offered by these plans. Fourth, our network breadth measure was constructed using the Ideon provider networks dataset, which has been used to assess physician networks in other studies.^27,34,35^ While Ideon implements quality assurance methods to minimize inaccuracies, the reliance on web-based scraping of health care practitioner directories may still introduce errors. However, any potential bias introduced by these data should be similar for both Asian-oriented affinity plans and other MA plans and thus unlikely to substantially affect the results. Additionally, our network breadth measure was based on physicians within plan counties and may underestimate the actual network breadth across counties, although this limitation again applies to both affinity and other MA plans. Fifth, only a small share of MA plans in this sample met our Asian-oriented affinity plan definition; therefore, some analyses may be underpowered and the estimates may be less precise. Nevertheless, we were able to detect significant differences in plan-benefit design and physician networks despite of the small exposure group. Finally, due to data limitations, we could assess only the size of the physician networks and were unable to examine whether Asian-oriented affinity plans were more likely to offer racial-concordant care.

## Conclusions

In this study, Asian Medicare beneficiaries disproportionately enrolled in MA Asian-oriented affinity plans, particularly in states with large Asian populations (California, New York, Texas, and Massachusetts). While these plans offered culturally relevant supplemental benefits and lower premiums, they also presented notable trade-offs, including reduced coverage of certain traditional benefits, lower performance on Medicare Star Ratings, and narrower physician networks. Policymakers should closely monitor the performance of Asian-oriented affinity plans to ensure that they improve care for Asian beneficiaries and that plan marketing strategies align with the health care needs of Medicare enrollees.

## Notes

Supplement 1. **eTable 1.** Number of Asian Affinity Plans Identified Using Alternative Methods **eFigure.** Select Features Highlighted on Asian Affinity Plan Websites, 2023 **eTable 2.** Plan Characteristics for Asian Affinity Plans vs. Other MA Plans, 2023 **eTable 3.** Adjusted Differences in Supplemental Benefits Between Asian Affinity Plans and Other MA Plans, 2023 **eTable 4.** CMS Star Rating Performance for Asian Affinity Plans vs. Other MA Plans, by Rating Domain, 2023 **eTable 5.** Adjusted Differences in Network Breadth Between Asian Affinity Plans vs. Other MA Plans, 2023 **eTable 6.** Adjusted Differences in Network Breadth Between Asian Affinity Plans vs. Other MA Plans, Excluding Plans with Extremely Narrow Networks, 2023

Supplement 2.
Data Sharing Statement
