# Supplementary material for: Benefit Design and Potential Trade-offs of Medicare Advantage Affinity Plans for Asian Beneficiaries
Source: JAMA Netw Open. 2025 Dec 16;8(12):e2548028. doi: 10.1001/jamanetworkopen.2025.48028 (PMC12709375; doi:10.1001/jamanetworkopen.2025.48028)
Supplement: Supplement 1. — eTable 1. Number of Asian Affinity Plans Identified Using Alternative Methods eFigure. Select Features Highlighted on Asian Affinity Plan Websites, 2023 eTable 2. Plan Characteristics for Asian Affinity Plans vs. Other MA Plans, 2023 eTable 3. Adjusted Differences in Supplemental Benefits Between Asian Affinity Plans and Other MA Plans, 2023 eTable 4. CMS Star Rating Performance for Asian Affinity Plans vs. Other MA Plans, by Rating Domain, 2023 eTable 5. Adjusted Differences in Network Breadth Between Asian Affinity Plans vs. Other MA Plans, 2023 eTable 6. Adjusted Differences in Network Breadth Between Asian Affinity Plans vs. Other MA Plans, Excluding Plans with Extremely Narrow Networks, 2023 [file jamanetwopen-e2548028-s001.pdf]

Supplemental Online Content

Ma Y, Hamed P, Stein RL, et al. Benefit design and potential tradeoffs of Medicare Advantage affinity plans for Asian beneficiaries. *JAMA Netw Open*. 2025;8(12):e2548028. doi:10.1001/jamanetworkopen.2025.48028

eTable 1. Number of Asian Affinity Plans Identified Using Alternative Methods

eFigure. Select Features Highlighted on Asian Affinity Plan Websites, 2023

eTable 2. Plan Characteristics for Asian Affinity Plans vs. Other MA Plans, 2023

eTable 3. Adjusted Differences in Supplemental Benefits Between Asian Affinity Plans and Other MA Plans, 2023

eTable 4. CMS Star Rating Performance for Asian Affinity Plans vs. Other MA Plans, by Rating Domain, 2023

eTable 5. Adjusted Differences in Network Breadth Between Asian Affinity Plans vs. Other MA Plans, 2023

eTable 6. Adjusted Differences in Network Breadth Between Asian Affinity Plans vs. Other MA Plans, Excluding Plans with Extremely Narrow Networks, 2023

This supplemental material has been provided by the authors to give readers additional information about their work.

© 2025 Ma Y et al. *JAMA Network Open*

eTable 1. Number of Asian Affinity Plans Identified Using Alternative Methods

		Alternative Method			
		Three Standard Deviation Method		Medicare Bayesian Improved Surname Geocoding Algorithm	
		Yes	No	Yes	No
**Main Method**	Yes	27	0	27	0
	No	15	4,224	0	4,228
**Sensitivity**		1		1	
**Specificity**		0.9965		1	
**Cohen's Kappa (95%CI)**		0.78 (0.67,0.89)		1 (1, 1)	

Notes:

1. In “three standard deviation method”, we classified an MA plan as an Asian affinity plans if the difference between its Asian enrollment and the Asian MA enrollment in its service area exceeded three standard deviations, which approximates the 99th percentile of the deviation from the Asian MA enrollment in the plan’s service area.
2. In “Medicare Bayesian Improved Surname Geocoding Algorithm Method”, we used Medicare Bayesian Improved Surname Geocoding algorithm instead of Research Triangle Institute race code to identify Asian beneficiaries and applied this alternative classification to define Asian affinity plans.
3. Total number of MA plans differed between the two alternative methods due to differences in the number of beneficiaries with missing race/ethnicity information between the Research Triangle Institute race code and the Medicare Bayesian Improved Surname Geocoding Algorithm, which affected the exclusion of MA plans with fewer than 100 enrollees.

© 2025 Ma Y et al. *JAMA Network Open*

eFigure. Select Features Highlighted on Asian Affinity Plan Websites, 2023

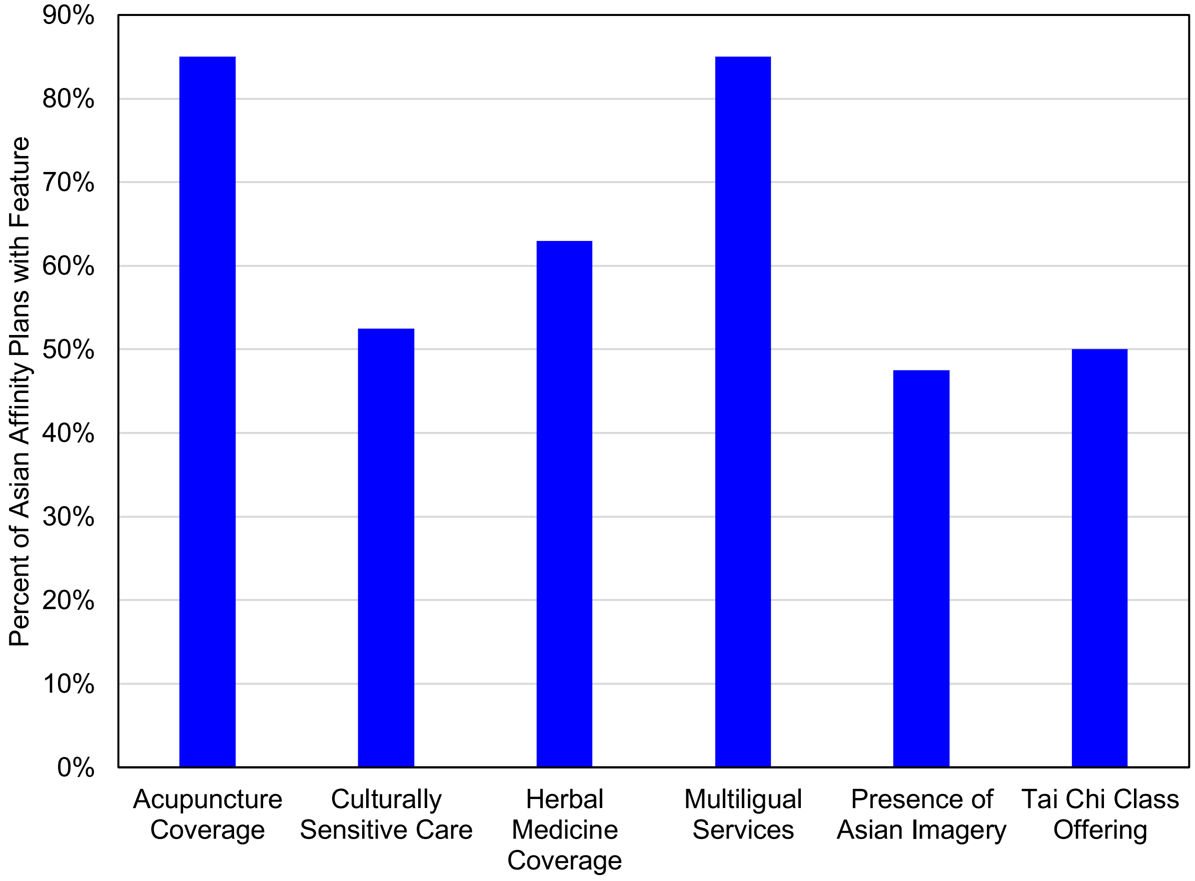

Notes:

1. For each Asian affinity plan, we manually reviewed its website and assessed the presence of Asian-focused marketing content for each of the categories listed above.

© 2025 Ma Y et al. *JAMA Network Open*

eTable 2. Plan Characteristics for Asian Affinity Plans vs. Other MA Plans, 2023

	Asian Affinity Plans	Other MA Plans	p value
Number of Plans	27	928	–
Total Enrollment	169,802	6,086,859	–
Total Asian Enrollment	109,906	574,858	–
% Asian Enrollees	64.7%	9.4%	–
Average Enrollment per Plan	6,289	6,559	–
Average Asian Enrollment per Plan	4,071	619	–
Plan Type			
PPO	11.3%	18.4%	0.358
HMO	88.7%	81.6%	
Special Needs Plan			
Yes	46.0%	25.4%	0.019
No	54.0%	74.6%	
Tax Status			
For Profit	92.6%	72.4%	<0.001
Not-for-Profit / Non-Profit	7.4%	27.6%	

Notes:

1. Analysis limited to MA plans offered within California, New York, Texas, and Massachusetts.
2. Unadjusted means for plan type and special needs plan status were compared using independent sample 2-tailed t test.
3. Analysis weighted by number of beneficiaries.

eTable 3. Adjusted Differences in Supplemental Benefits Between Asian Affinity Plans and Other MA Plans, 2023

Supplemental Benefits	Asian Affinity Plans	Other MA Plans	Adjusted Difference	95% CI
Acupuncture	86.2%	63.0%	23.2%	(23.0%,23.4%)
Alternative therapies	10.6%	5.8%	4.8%	(4.7%,5.0%)
Annual physical exam	53.3%	95.0%	-41.7%	(-41.9%,-41.5%)
Chiropractic services	21.9%	26.6%	-4.7%	(-4.9%,-4.5%)
Comprehensive dental items	85.1%	92.6%	-7.6%	(-7.7%,-7.4%)
Eyewear	94.9%	98.9%	-3.9%	(-4.0%,-3.8%)
Hearing aids	95.1%	92.9%	2.2%	(2.1%,2.2%)
Hearing exams	95.9%	94.2%	1.7%	(1.7%,1.8%)
In-home support services	59.2%	16.0%	43.2%	(42.9%,43.4%)
Over-the-counter (OTC) items	99.3%	89.5%	9.8%	(9.8%,9.9%)
Podiatry services	22.1%	45.6%	-23.6%	(-23.8%,-23.4%)
Preventive dental items	96.0%	93.9%	2.2%	(2.1%,2.2%)
Remote access technologies	90.2%	82.7%	7.5%	(7.4%,7.7%)
Transportation services	75.5%	51.6%	23.9%	(23.7%,24.1%)
Worldwide emergency/urgent coverage	100.0%	98.5%	1.5%	(1.5%,1.5%)

Notes:

1. Analysis limited to MA plans offered within California, New York, Texas, and Massachusetts.
2. Adjusted means and differences in supplemental benefit coverage between Asian affinity plans and other MA plans were estimated using enrollee-weighted linear regression models. The dependent variable indicated whether a plan offered the supplemental benefit of interest, and explanatory variables included an indicator for Asian affinity plans, plan type, and special needs plan status.

© 2025 Ma Y et al. *JAMA Network Open*

eTable 4. CMS Star Rating Performance for Asian Affinity Plans vs. Other MA Plans, by Rating Domain, 2023

	Affinity Plans		Control Plans				
Star Ratings	N	Unadjusted Mean	N	Unadjusted Mean	Unadjusted Difference	Adjusted Difference	Adjusted 95% CI
**Overall**	16	3.27	842	4.14	-0.87	-0.73	(-1.04,-0.42)
					0.00		
**Part C**	16	3.19	842	4.03	-0.84	-0.69	(-0.97,-0.42)
HD1: Staying Healthy: Screenings, Tests and Vaccines	16	3.70	830	4.29	-0.59	-0.56	(-0.83,-0.28)
HD2: Managing Chronic (Long Term) Conditions	16	3.57	842	4.02	-0.46	-0.43	(-0.64,-0.21)
HD3: Member Experience with Health Plan	20	1.68	818	3.22	-1.54	-1.32	(-1.66,-0.98)
HD4: Member Complaints and Changes in the Health Plan's Performance	20	3.50	842	4.18	-0.68	-0.66	(-0.99,-0.33)
HD5: Health Plan Customer Service	13	4.64	800	4.76	-0.12	-0.05	(-0.3,0.21)
**Part D**	25	3.39	860	4.05	-0.66	-0.64	(-0.94,-0.34)
DD1: Drug Plan Customer Service	27	4.35	902	4.74	-0.39	-0.35	(-0.6,-0.11)
DD2: Member Complaints and Changes in the Drug Plan’s Performance	21	3.50	844	4.06	-0.56	-0.63	(-0.95,-0.3)
DD3: Member Experience with the Drug Plan	11	3.05	756	3.90	-0.85	-1.04	(-1.78,-0.3)
DD4: Drug Safety and Accuracy of Drug Pricing	25	3.34	860	3.94	-0.60	-0.62	(-0.86,-0.38)

Notes:

1. Analysis limited to MA plans offered within California, New York, Texas, and Massachusetts.
2. Adjusted differences between Asian affinity plans and other MA plans were estimated using enrollee-weighted linear regression models. The dependent variable is the measure of interest, and explanatory variables included an indicator for Asian affinity plans, plan type, special needs plan status, and state fixed effects.
3. Both unadjusted and adjusted analyses are weighted by number of beneficiaries.

© 2025 Ma Y et al. *JAMA Network Open*

eTable 5. Adjusted Differences in Network Breadth Between Asian Affinity Plans vs. Other MA Plans, 2023

	Number of Plans		Adjusted Network Breadth Difference Relative to Other MA Plans					
			Overall		Primary Care		Specialist	
	Asian Affinity Plans	Other MA Plans	Estimate	S.E.	Estimate	S.E.	Estimate	S.E.
Overall	27	928	0.0002	0.09	-0.07	0.07	0.02	0.09
California	21	388	-0.13***	0.04	-0.16****	0.04	-0.12***	0.04
Non-California	6	540	0.06	0.09	-0.03	0.08	0.09	0.10

Notes:

1. Analysis limited to MA plans offered within California, New York, Texas, and Massachusetts.
2. Adjusted differences in network breadth between Asian affinity plans and other MA plans were estimated using enrollee-weighted linear regression models. The dependent variable was the network breadth of the plan, and explanatory variables included an indicator for Asian affinity plans, plan type, special needs plan status, and state fixed effects.
3. Network breadth calculated at the plan-county level as the proportion of in-network physicians in a county out of the total number of physicians in network for at least one plan of analysis in that county. Network breadth at the plan level was then calculated as a beneficiary-weighted average of plan-county breadths.
4. *p<0.1, **p<0.05, ***p<0.01, ****p<0.001

© 2025 Ma Y et al. *JAMA Network Open*

eTable 6. Adjusted Differences in Network Breadth Between Asian Affinity Plans vs. Other MA Plans, Excluding Plans with Extremely Narrow Networks, 2023

	Number of Plans		Adjusted Network Breadth Difference Relative to Other MA Plans					
			Overall		Primary Care		Specialist	
	Asian Affinity Plans	Other MA Plans	Estimate	S.E.	Estimate	S.E.	Estimate	S.E.
Overall	23	880	-0.02	0.08	-0.10	0.06	0.00	0.08
California	17	373	-0.13***	0.05	-0.17****	0.05	-0.12***	0.04
Non-California	6	507	0.02	0.09	-0.08	0.08	0.04	0.09

Notes:

1. Analysis limited to MA plans offered within California, New York, Texas, and Massachusetts.
2. Plans with network breadth in the lowest 5th percentile were excluded.
3. Adjusted differences in network breadth between Asian affinity plans and other MA plans were estimated using enrollee-weighted linear regression models. The dependent variable was the network breadth of the plan, and explanatory variables included an indicator for Asian affinity plans, plan type, special needs plan status, and state fixed effects.
4. Network breadth calculated at the plan-county level as the proportion of in-network physicians in a county out of the total number of physicians in network for at least one plan of analysis in that county. Network breadth at the plan level was then calculated as a beneficiary-weighted average of plan-county breadths.
5. *p<0.1, **p<0.05, ***p<0.01, ****p<0.001

© 2025 Ma Y et al. *JAMA Network Open*
