# Supplementary material for: Benefit Design and Potential Trade-offs of Medicare Advantage Affinity Plans for Asian Beneficiaries
Source: JAMA Netw Open. 2025 Dec 16;8(12):e2548028. doi: 10.1001/jamanetworkopen.2025.48028 (PMC12709375; doi:10.1001/jamanetworkopen.2025.48028)
Supplement: Supplement 2. — Data Sharing Statement [file jamanetwopen-e2548028-s002.pdf]

Data Sharing Statement

Ma. Benefit Design and Potential Tradeoffs of Medicare Advantage Affinity Plans for Asian Beneficiaries. *JAMA Netw Open*. Published December 16, 2025. doi:10.1001/jamanetworkopen.2025.48028

Data
Data available: No
